# TGF-β/Smad signaling in renal fibrosis

**DOI:** 10.3389/fphys.2015.00082

**Published:** 2015-03-19

**Authors:** Xiao-Ming Meng, Patrick Ming-Kuen Tang, Jun Li, Hui Yao Lan

**Affiliations:** ^1^School of Pharmacy, Anhui Medical UniversityHefei, China; ^2^Department of Medicine and Therapeutics, The Chinese University of Hong KongHong Kong, China; ^3^Li Ka Shing Institute of Health Sciences, The Chinese University of Hong KongHong Kong, China; ^4^Shenzhen Research Institute, The Chinese University of Hong KongShenzhen, China

**Keywords:** TGF-ß, Smads mediators, renal fibrosis, therapeutics, mechanisms

## Abstract

TGF-β (transforming growth factor-β) is well identified as a central mediator in renal fibrosis. TGF-β initiates canonical and non-canonical pathways to exert multiple biological effects. Among them, Smad signaling is recognized as a major pathway of TGF-β signaling in progressive renal fibrosis. During fibrogenesis, Smad3 is highly activated, which is associated with the down-regulation of an inhibitory Smad7 via an ubiquitin E3-ligases-dependent degradation mechanism. The equilibrium shift between Smad3 and Smad7 leads to accumulation and activation of myofibroblasts, overproduction of ECM (extracellular matrix), and reduction in ECM degradation in the diseased kidney. Therefore, overexpression of Smad7 has been shown to be a therapeutic agent for renal fibrosis in various models of kidney diseases. In contrast, another downstream effecter of TGF-β/Smad signaling pathway, Smad2, exerts its renal protective role by counter-regulating the Smad3. Furthermore, recent studies demonstrated that Smad3 mediates renal fibrosis by down-regulating miR-29 and miR-200 but up-regulating miR-21 and miR-192. Thus, overexpression of miR-29 and miR-200 or down-regulation of miR-21 and miR-192 is capable of attenuating Smad3-mediated renal fibrosis in various mouse models of chronic kidney diseases (CKD). Taken together, TGF-β/Smad signaling plays an important role in renal fibrosis. Targeting TGF-β/Smad3 signaling may represent a specific and effective therapy for CKD associated with renal fibrosis.

## Introduction

The TGF-β superfamily consists of highly pleiotropic molecules including activins, inhibins, BMPs (Bone morphogenic proteins), GDFs (Growth differentiation factors) and GDNFs (Glial-derived neurotrophic factors), and exerts multiple biological functions in renal inflammation, fibrosis, cell apoptosis, and proliferation (Massague and Wotton, 2000). Among them, TGF-β is known as a key pro-fibrotic mediator in fibrotic diseases. Three isoforms of TGF-β have been identified in mammals, termed TGF-β1, 2, and 3, of which TGF-β1 is the most abundant isoform and can be produced by all types of renal resident cells. After synthesis, TGF-β1 is released in association with LAP (latency-associated peptide) as a latent form of TGF-β1 which binds to LTBP (Latent TGF-β-binding protein) in the target tissues. When exposed to multiple types of stimuli, including ROS (Reactive oxygen species), plasmin and acid (Lyons et al., 1990; Munger et al., 1999; Meng et al., 2013), TGF-β1 can be released from the LAP and LTBP and becomes active. The active TGF-β1 then binds to TβRII (Type II TGF-β receptor), a constitutively active kinase, which recruits TβRI (Type I TGF-β receptor) and phosphorylates the downstream receptor-associated Smads (R-Smads) i.e., Smad2 and Smad3 (Wrana et al., 1994). Then the phosphorylated Smad2 and Smad3 form an oligomeric complex with a common Smad, Smad4, and translocates into the nucleus to regulate the transcription of target genes in collaboration with various co-activators and co-repressors. It is interesting that an inhibitory Smad, Smad7, can be induced in a Smad3-dependent manner. Smad7 consequently competes with the R-Smads for binding to the activated receptors, in order to exert its negative effect on TGF-β/Smad signaling (Shi and Massague, 2003). Additionally, TGF-β1 is able to function through the Smad-independent pathways, including p38, ERK (Extracellular-signal-regulated kinase), MAPK, Rho-GTPases, Rac, Cdc42, ILK (Integrin linked kinase) (Attisano and Wrana, 2002; Derynck and Zhang, 2003; Li et al., 2009; Loeffler and Wolf, 2014). In this review, we focus on the pathological roles of TGF-β/Smad signaling in renal fibrosis.

## Role of TGF-β1 in renal fibrosis

**Figure 1:**
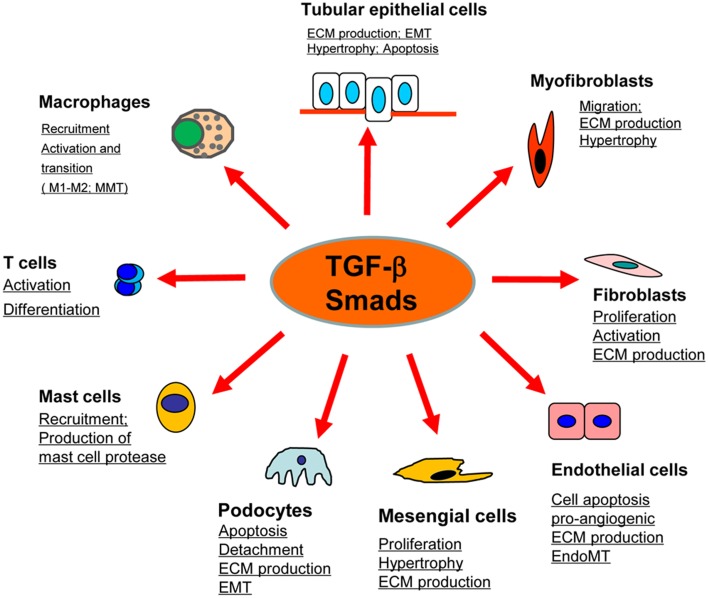
**Role of TGF-β/Smad signaling in kidney disease**. TGF-β1 signals through the downstream mediators to exert its biological activities on different cell types of kidney cells during renal inflammation and fibrosis.

Renal fibrosis, characterized by excessive deposition of ECM (Extracellular matrix), is recognized as a common pathological feature of CKD (Chronic kidney diseases) which leads to the development of ESRD (End-stage renal disease), accompanied by a progression of renal malfunctions (Eddy and Neilson, 2006). Although effective therapy for renal fibrosis is still lacking, a number of studies demonstrated that TGF-β is the key mediator in CKD associated with progressive renal fibrosis. It is well documented that TGF-β1 has multiple biological properties including cell proliferation, differentiation, apoptosis, autophagy, production of ECM, etc. (Meng et al., 2013). Considerable evidence revealed that TGF-β is substantially upregulated in the injured kidney on both patients and animal disease models (Yamamoto et al., 1996; Bottinger and Bitzer, 2002). It is also showed that the urinary levels of TGF-β are significantly increased in patients with various renal diseases, which is positively correlated with the degree of renal fibrosis (Murakami et al., 1997). Moreover, the importance of TGF-β1 in renal fibrosis is further supported by the findings that overexpression of active TGF-β1 in rodent liver is capable of inducing the fibrotic response in kidney; whereas blocking TGF-β with neutralizing antibody, antisense oligonucleotides, inhibitors, or genetic deletion of receptors can attenuate kidney fibrosis *in vivo* and *in vitro* (Sanderson et al., 1995; Kopp et al., 1996; Border and Noble, 1998; Moon et al., 2006; Petersen et al., 2008; Meng et al., 2012a). In contrast to the active form of TGF-β1, the latent form of TGF-β1 can protect the kidney against fibrosis and inflammation by upregulating Smad7 that is observed in the latent TGF-β transgenic mice received with UUO-induced nephropathy or anti-GBM-induced glomerulonephritis (Huang et al., 2008a,b). Taken together, TGF-β exerts profibrotic effects on the kidney through several possible mechanisms: (1) TGF-β1 directly induces the production of ECM, including collagen I and fibronectin, through the Smad3-dependent or -independent mechanisms (Samarakoon et al., 2012); (2) TGF-β1 suppresses the degradation of ECM by inhibiting MMPs (Matrix metalloproteinases) but inducing TIMPs (Tissue inhibitor of metalloproteinase) and the natural inhibitor of MMPs; (3) TGF-β1 is believed to play critical roles in the transdifferentiation toward myofibroblast of several types of cells, including epithelial cells, endothelial cells, and pericytes, although the origin of myofibroblast is still undefined (Meng et al., 2013; Wu et al., 2013); (4) TGF-β1 acts directly on different types of renal resident cells, for example: it can promote the proliferation of mesangial cells in order to increase matrix production, or induce the elimination of tubular epithelial cells and podocytes which may lead to a deterioration of renal injury and incur more severe renal fibrosis (Bottinger and Bitzer, 2002; Lopez-Hernandez and Lopez-Novoa, 2012) (Figure 1).

## Role of Smads in renal fibrosis

### Smad2 and Smad3

It is consistently demonstrated that Smad2 and Smad3 are extensively activated in the fibrotic kidney in patients and animal models with CKD (Meng et al., 2013). Although Smad2 and Smad3 share more than 90% similarity in their amino acid sequences, their functional roles in renal fibrosis are distinct. It is well documented that Smad3 is pathogenic since knockout of Smad3 gene inhibits fibrosis in obstructive nephropathy (Sato et al., 2003), diabetic nephropathy (Fujimoto et al., 2003), hypertensive nephropathy (Liu et al., 2012), and drug-toxicity-related nephropathy (Zhou et al., 2010). Of note, Smad3 promotes renal fibrosis by directly binding to the promoter region of collagens to trigger their production (Vindevoghel et al., 1998; Chen et al., 1999), and inhibiting the ECM degradation via induction of TIMP-1 while reducing MMP-1 activities in fibroblasts (Yuan and Varga, 2001). In contrast to Smad3, Smad2 is unable to directly bind to the genomic DNA (Dennler et al., 1998). Previous study suggested that roles of Smad2 and Smad3 might be different in fibrotic diseases (Piek et al., 2001; Yang et al., 2003b; Phanish et al., 2006). Consistent with the finding that the endogenous ratio of Smad2 and Smad3 may ultimately influence the cytostatic function of Smad3 (Kim et al., 2005), results from our recent study demonstrated that conditional knockout of Smad2 from tubular epithelial cells enhances Smad3-mediated renal fibrosis *in vivo* and *in vitro*, which is associated with the increase in phosphorylation and nuclear translocation of Smad3, promotion of the Smad3 responsive promoter activity, and binding of Smad3 to Col1A2 promoter (Meng et al., 2010).

### Smad4

As a common Smad for TGF-β/BMP signaling, Smad4 plays a critical role in nucleocytoplasmic shuttling of Smad2/3 and Smad1/5/8 complexes (Massague and Wotton, 2000). It has been demonstrated that loss of Smad4 in mesangial cells inhibits TGF-β1-induced ECM deposition (Tsuchida et al., 2003), which is further confirmed by our recent finding that specific deletion of Smad4 from renal tubular epithelial cells attenuates the UUO-induced renal fibrosis by suppressing Smad3 responsive promoter activity and decreasing the binding of Smad3 to the target genes independent of its phosphorylation and nuclear translocation (Meng et al., 2012b).

### Smad7

As an inhibitory regulator in the TGF-β/Smad signaling pathway, Smad7 can be induced by a Smad3-dependent mechanism, which in turn blocks the signal transduction of TGF-β1 via its negative feedback loop (Afrakhte et al., 1998; Zhu et al., 1999; Kavsak et al., 2000; Ebisawa et al., 2001). Moreover, the regulatory mechanism of Smad7 on TGF-β signaling occurs in an elegant manner, i.e., TGF-β not only induces Smad7 transcription, but also promotes the degradation of Smad7 by activating the Smad3-dependent Smurfs/arkadia-mediated ubiquitin–proteasome degradation pathway (Kavsak et al., 2000; Ebisawa et al., 2001; Fukasawa et al., 2004; Liu et al., 2008). In this setting, the level of Smad7 protein is significantly reduced in response to the high level of active TGF-β1 in CKD. Most importantly, the functional role of Smad7 is further defined by the findings that deletion of Smad7 accelerates renal fibrogenesis in obstructive nephropathy, diabetic nephropathy as well as hypertensive nephropathy (Chung et al., 2009; Chen et al., 2011; Liu et al., 2013), suggesting Smad7 as a therapeutic agent for treatment of CKD (Lan et al., 2003; Hou et al., 2005; Ka et al., 2007, 2012; Chen et al., 2011; Liu et al., 2014).

Collectively, compelling evidence indicates that hyperactivation of Smad3 associated with progressive degradation of Smad7, is a key feature of renal fibrotic diseases. More importantly, the imbalance of Smad3 and Smad7 was proved to be one of the major mechanisms in mediating the fibrotic response. In this regard, rebalancing the disturbed Smad3/Smad7 ratio, through downregulating Smad3 and upregulating Smad7 simultaneously, seems to be an effective strategy for treatment of renal fibrosis.

## Role of TGF-β in transdifferentiation of myofibroblasts

Emerging evidence suggests that the accumulation of myofibroblasts, a predominant source for ECM production, is a critical step in the progression of renal fibrosis (Wynn and Ramalingam, 2012). However, the origin of myofibroblast is still controversial. It has been reported that myofibroblasts may be derived from the resident fibroblasts, pericytes, bone marrow cells (e.g., fibrocytes), epithelial cells (Epithelial–mesenchymal transition, EMT), and endothelial cells (Endothelial–mesenchymal transition, EndMT) (Allison, 2013; LeBleu et al., 2013; Meng et al., 2014). Our latest data also revealed that bone marrow-derived macrophages were capable of becoming myofibroblast phenotype via a process of macrophage-myofibroblast transition (MMT) in patients and UUO model with active renal fibrosis (Nikolic-Paterson et al., 2014). In addition, it is generally accepted that local fibroblasts can differentiate into myofibroblast under the stimulation of TGF-β (Evans et al., 2003; Midgley et al., 2013). Increasing evidence indicates that fibrocytes can produce a large amount of collagens directly in response to the stimulus such as TGF-β (Hong et al., 2007; Wada et al., 2007). Administration of TGF-β promotes the transdifferentiation of epithelial cells and endothelial cells toward myofibroblast-like cells, whereas, blockade of TGF-β/Smad signaling with inhibitors or antagonists attenuates or reverses the process of EMT or EndMT (Fan et al., 1999; Zeisberg et al., 2003, 2008; Li et al., 2010; Liu, 2010; Yang et al., 2010; Xavier et al., 2014). In addition, TGF-β1 can promote renal fibrosis via the cell-cell interaction mechanism as TGF-β1 released from the injured epithelium is able to activate pericyte-myofibroblast transition (Wu et al., 2013). Moreover, we also identify that advanced glycation end products (AGEs) and angiotensin II are capable of activating Smad3 to mediate the process of EMT under diabetes and hypertension conditions (Li et al., 2003, 2004; Wang et al., 2006b; Yang et al., 2009, 2010; Chung et al., 2010b).

## Role of TGF-β1/Smad-dependent miRNAs in renal fibrosis

**Figure 2:**
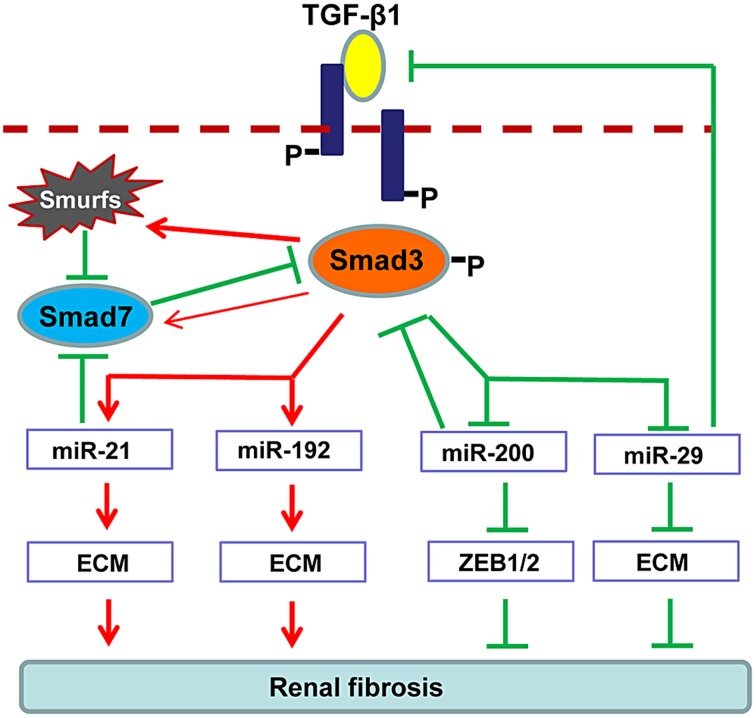
**Regulation of TGF-β/Smad3 in fibrosis-related microRNAs during renal fibrosis**. TGF-β1 activates Smad3 that binds directly to a number of microRNAs to either negatively or positively regulate their expression and function in renal fibrosis.

Increasing evidence demonstrates that TGF-β1 can also regulate several miRNAs to facilitate renal fibrogenesis. As illustrated in Figure 2, TGF-β1 up-regulates miR-21, miR192, miR-377, miR-382, and miR-491-5p, but down-regulates miR-29 and miR-200 families during renal fibrosis (Kantharidis et al., 2011; Kriegel et al., 2012; Lan and Chung, 2012; Chung et al., 2013a,b). In fibrotic kidneys, the level of miR-21 is highly induced (Godwin et al., 2010; Zhong et al., 2011, 2013; Chau et al., 2012; Xu et al., 2012; Wang et al., 2013), whereas inhibition of miR-21 attenuates deposition of ECM and halts the progression of renal fibrosis (Zhong et al., 2011, 2013; Chau et al., 2012). Role of miR-192 in fibrosis is still controversial. It is reported that miR-192 is elevated in fibrotic mouse models and TGF-β1-treated murine cells (Kato et al., 2007; Chung et al., 2010a; Putta et al., 2012). Knockout or knockdown of miR-192 largely attenuated renal fibrosis possibly through induction of ZEB1/2 *in vivo* and *in vitro*. However, a recent study indicated that TGF-β1 reduces miR-192 expression in human TECs and deficiency of miR-192 accelerates renal fibrosis in diabetic nephropathy (Krupa et al., 2010), which is further evident by the results from the renal biopsy of diabetic patients with lower level of miR-192 (Wang et al., 2010). The discrepancy in these studies suggests the complexity of miR-192 in renal fibrogenesis. The miR-29 and miR-200 are TGF-β1-dependent anti-fibrotic miRNAs that are extensively suppressed in the diseased kidneys (Qin et al., 2011). Of note, more than 20 ECM-related genes, including collagens, are potential targets for miR-29 where some of them are regulated by the TGF-β signaling (van Rooij et al., 2008; Xiao et al., 2012). Overexpression of miR-29 attenuates renal fibrosis *in vivo* in obstructive and diabetic nephropathies and suppresses the fibrotic genes *in vitro* in response to various stimuli including TGF-β1, high glucose or salt-induced hypertensive conditions (Du et al., 2010; Liu et al., 2010; Qin et al., 2011; Chen et al., 2014). The miR-200 family contains miR-200a, miR-200b, miR-200c, miR-429, and miR-141 (Howe et al., 2012). Downregulation of miR-200a and miR-141 are detected in the fibrotic kidneys of obstructive and diabetic nephropathies (Wang et al., 2011; Xiong et al., 2012). As miR-200 has a major role in maintaining the epithelial differentiation, delivery of miR-200b significantly reduces renal fibrotic response by suppressing the transcriptional repressors of E-cadherin ZEB1 and ZEB2 (Korpal et al., 2008; Oba et al., 2010).

## TGF-β/Smad signaling as a therapeutic potential for renal fibrosis

### General blockade of TGF-β signaling

The therapeutic potential of anti-TGF-β1 therapy has been widely tested according to the pathogenic role of TGF-β1 in fibrogenesis. It has been shown that TGF-β neutralizing antibodies, antisense TGF-β oligodeoxynucleotides, soluble human TβRII (sTβRII.Fc) and specific inhibitors to TβR kinases (such as GW788388 and IN-1130) can effectively halt the progression of renal fibrosis in a number of experimental kidney disease models. A recent study also demonstrated that blockade of TGF-β1 receptor posttranslational core fucosylation can attenuate renal interstitial fibrosis (Shen et al., 2013). In addition, some TGF-β inhibitors have been further tested in preclinical or clinical trials (Tampe and Zeisberg, 2014). For an instance, treatment with Pirfenidone, a small molecule that blocks TGF-β1 promoter, can prevent the decline of eGFR (estimated glomerular filtration rate) in patients with focal segmental glomerulosclerosis (FSGS) or diabetic nephropathy (Cho et al., 2007; Sharma et al., 2011). In addition, Fresolimumab and LY2382770, neutralizers for TGF-β1 activity, are also tested in FSGS and diabetic kidney diseases in human (Trachtman et al., 2011; Choi et al., 2012; Tampe and Zeisberg, 2014). However, the major obstacle and risk for these potential therapies by generally blocking TGF-β signaling may be related to the abrogation of its anti-inflammatory and anti-tumorigenesis property. Nevertheless, it should be mentioned that TGF-β1 may also serve as a potential biomarker for renal fibrosis, since significant upregulation of urine TGF-β1 have been detected in progressive renal diseases (Tsakas and Goumenos, 2006).

### Specific inhibition of downstream Smads or Smad-regulated miRNAs

**Figure 3:**
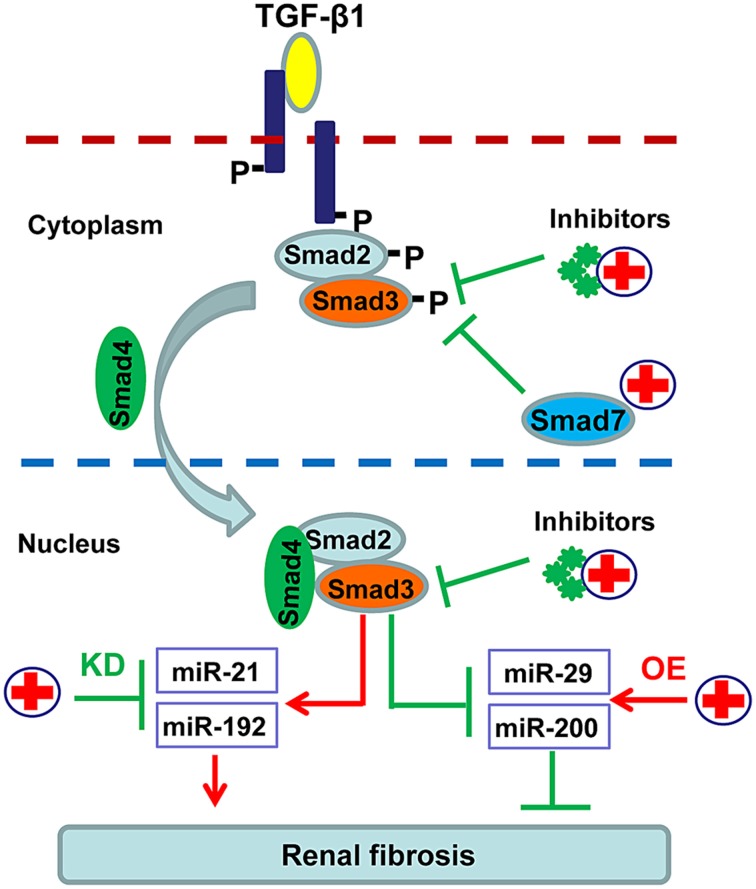
**Potential therapeutic strategies for renal fibrosis by specifically targeting downstream TGF-β/Smad signaling**. Since renal fibrosis is mediated positively by Smad3 but negatively by Smad7, treatment for renal fibrosis can target Smad3 with specific inhibitors or Smad3-dependent microRNAs that regulate fibrosis, and/or by promoting Smad7 with gene therapy or specific agonists.

In order to avoid the side effects caused by complete blockade of TGF-β1 signaling, more focus has been paid on inhibiting the downstream targets of this signaling pathway including Smad3, Smad7, and Smad-dependent miRNAs (Ng et al., 2009). As shown in Figure 3, SIS3, a specific inhibitor of Smad3 phosphorylation, can attenuate renal fibrosis in diabetic nephropathy (Li et al., 2010). Accumulated evidence shows that targeting Smad3 by overexpressing renal Smad7 produces inhibitory effects on both renal inflammation and fibrosis in a variety of kidney disease models (Hou et al., 2005; Ka et al., 2007, 2012; Chen et al., 2011; Liu et al., 2014). Moreover, recent studies also revealed that overexpression of miR-29, miR-200 or inhibition of miR-21 and miR-192 can effectively decelerate the progression of renal fibrosis (Oba et al., 2010; Chung et al., 2010a; Qin et al., 2011; Zhong et al., 2011, 2013; Chen et al., 2014) (Figure 3).

BMP-7 is a natural antagonist for TGF-β through inhibiting the TGF-β/Smad3, which has been demonstrated on various renal disease models (Hruska et al., 2000; Zeisberg et al., 2003; Wang et al., 2006a; Sugimoto et al., 2007; Luo et al., 2010; Meng et al., 2013). Klotho, a single-pass transmembrane protein predominantly expressed in renal tubular epithelial cells, is capable of suppressing renal fibrosis by directly binding to type II TGF-β receptor to block the TGF-β-initiated signaling (Doi et al., 2011). Most recently, a study showed that an adaptor protein, Kindlin-2, recruits Smad3 to TGF-β type I receptor, therefore contributing to TGF-β/Smad3-mediating renal interstitial fibrosis (Wei et al., 2013). In addition, two well-known Smad transcriptional co-repressors Ski (Sloan-Kettering Institute proto-oncogene) and SnoN (Ski-related novel gene, non Alu-containing), elicit their anti-fibrotic effects on TGF-β by antagonizing Smad-mediated gene transcription (Yang et al., 2003a). Moreover, GQ5, a small molecular phenolic compound extracted from dried resin of *Toxicodendron vernicifluum*, has been shown to inhibit the interaction between TGF-β type I receptor and Smad3 through interfering the binding of Smad3 to SARA, thereby reducing the phosphorylation of Smad3 and downregulating the transcription of downstream fibrotic indexes including α-SMA, collagen I and fibronectin *in vivo* and *in vitro* (Ai et al., 2014). Furthermore, a number of miRNAs, such as let-7b and miR-29, are capable of regulating TGF-β signaling and altering the progression of renal fibrosis (Kato et al., 2011; Xiao et al., 2012; Wang et al., 2014).

## Conclusion

An equilibrium shift of TGF-β/Smad signaling due to the hyperactivation of Smad3 but reduction of Smad7 may be a key pathological mechanism leading to renal fibrogenesis. Thus, rebalancing the TGF-β/Smad signaling by targeting Smad3 activity, up-regulating Smad7, as well as specifically modulating Smad3-dependent miRNAs related to fibrosis may represent an effective therapy for CKD associated with progressive real fibrosis.

### Conflict of interest statement

The authors declare that the research was conducted in the absence of any commercial or financial relationships that could be construed as a potential conflict of interest.
